# Mental health in time of pandemics: Study protocol to incorporate risk and protective factors contributing to psychological stress among portuguese and swiss higher education students

**DOI:** 10.1192/j.eurpsy.2021.1218

**Published:** 2021-08-13

**Authors:** C. Laranjeira, A. Querido, O. Valentim, Z. Charepe, M. Dixe

**Affiliations:** 1 Citechcare, Polytechnic of Leiria, Leiria, Portugal; 2 School Of Health Sciences, Polytechnic of Leiria, Leiria, Portugal; 3 Instituto De Ciências Da Saúde, Universidade Católica Portuguesa, Lisboa, Portugal

**Keywords:** Study protocol, pandemics, psychological stress, higher education students

## Abstract

**Introduction:**

The ongoing COVID-19 pandemic is inducing fear, and a timely understanding of mental health status is urgently needed for society. Previous research has revealed a profound and wide range of psychosocial impacts on people at the individual, community, and international levels. On an individual level, people are likely to experience fear of falling sick or dying themselves, feelings of helplessness, and stigma. Currently, there is little understanding of mental well-being assessment under scenarios of pandemics that oblige to social isolation and quarantine.

**Objectives:**

This study aims to: a) establish the prevalence of psychiatric symptoms; b) identify risk and protective factors contributing to psychological stress; and c) identify coping strategies to promote better adjustment during and after the pandemic crisis.

**Methods:**

We will adopt a mixed-method approach, firstly with a cross-sectional survey design (in both Portugal and Swiss context) to assess the higher education student’s psychosocial response during and after the pandemic, by using an anonymous online questionnaire. In a 2nd phase, and in order to gain more insight into the psychological stress faced by the students as a result of pandemic, a qualitative approach was chosen, focusing on the experiences of the participants.

**Results:**

This study has received ethical approval from both international and local institutional review boards. Data collection will start in November 2020 and will be completed at February 2021.

**Conclusions:**

The findings of this study will provide important data to assist government agencies and healthcare professionals in safeguarding the psychosocial wellbeing of the community in the face of COVID-19 outbreak expansion.

